# Supplementary material for: Glucose Fluctuation and Severe Internal Carotid Artery Siphon Stenosis in Type 2 Diabetes Patients
Source: Nutrients. 2021 Jul 12;13(7):2379. doi: 10.3390/nu13072379 (PMC8308661; doi:10.3390/nu13072379)
Supplement: Supplementary file 1 [file nutrients-13-02379-s001.zip › nutrients-1285219-supplementary.pdf]

Supplementary Material

Table S1. Baseline patient characteristics by degree of internal carotid artery siphon stenosis.

	Intracranial internal carotid artery siphon stenosis		
**Variables**	**Moderate (*n* = 48)**	**Mild (*n* = 47)**	***p* value**
Baseline demographics			
Female (%)	12 (25)	14 (30)	0.65
Age, years	77 ± 5	76 ± 6	0.32
Cerebrovascular risk factors			
Hypertension (%)	46 (96)	42 (89)	0.27
Dyslipidemia (%)	42 (88)	41 (87)	1.00
Current or past smoking (%)	28 (58)	26 (55)	0.84
Atrial fibrillation (%)	10 (21)	6 (13)	0.41
Medical history of percutaneous coronary intervention or coronary artery bypass grafting (%)	23 (48)	18 (38)	0.41
Ischemic stroke episode (%)	19 (40)	23 (49)	0.41
Diabetes mellitus			
Duration, years	23 ± 11	22 ± 13	0.47
HbA1c at registration, %	7.5 ± 1.0	7.5 ± 0.8	0.33
Blood glucose at registration, mg/dL	149 ± 49	152 ± 35	0.43
Blood glucose (CGM average), mg/dL	135 ± 27	138 ± 30	0.67
SD, mg/dL	40 ± 12	38 ± 9	0.63
%CV	29 ± 7	28 ± 5	0.50
MAGE, mg/dL	91 ± 26	89 ± 19	0.76

Continuous variables are shown as the mean (± SD), while categorical variables are shown as frequencies and percentages. Abbreviations: HbA1c, hemoglobin A1c; CGM, continuous glucose monitoring; SD, standard deviation; %CV, coefficient of variation; MAGE, mean amplitude of glycemic excursions.

Table S2. Baseline patient characteristics by degree of middle cerebral artery stenosis

	M1 portion of the middle cerebral artery stenosis		
**Variables**	**Moderate (*n* = 3)**	**Mild (*n* = 100)**	***p* value**
Baseline demographics			
Female (%)	0 (0)	32 (32)	0.55
Age, years	74 ± 6	76 ± 5	0.46
Cerebrovascular risk factors			
Hypertension (%)	3 (100)	91 (91)	1.00
Dyslipidemia (%)	3 (100)	88 (88)	1.00
Current or past smoking (%)	2 (67)	55 (55)	1.00
Atrial fibrillation (%)	0 (0)	17 (17)	1.00
Medical history of percutaneous coronary intervention or coronary artery bypass grafting (%)	1 (33)	44 (44)	1.00
Ischemic stroke episode (%)	1 (33)	44 (44)	1.00
Diabetes mellitus			
Duration, years	17 ± 10	23 ± 12	0.44
HbA1c at registration, %	7.9 ± 0.6	7.5 ± 0.9	0.23
Blood glucose at registration, mg/dL	141 ± 32	150 ± 43	0.75
Blood glucose (CGM average), mg/dL	123 ± 29	138 ± 28	0.36
SD, mg/dL	34 ± 13	40 ± 11	0.39
%CV	27 ± 4	29 ± 6	0.62
MAGE, mg/dL	80 ± 27	92 ± 23	0.37

Continuous variables are shown as the mean (± SD), while categorical variables are shown as frequencies and percentages. Abbreviations: HbA1c, hemoglobin A1c; CGM, continuous glucose monitoring; SD, standard deviation; %CV, coefficient of variation; MAGE, mean amplitude of glycemic excursions.
